# 2-[3,5-Bis­(4-fluoro­phen­yl)-4,5-dihydro-1*H*-pyrazol-1-yl]-4,6-bis(4-fluoro­phenyl)pyrimidine

**DOI:** 10.1107/S1600536812006976

**Published:** 2012-02-24

**Authors:** Hoong-Kun Fun, Tze Shyang Chia, S. Samshuddin, B. Narayana, B. K. Sarojini

**Affiliations:** a X-ray Crystallography Unit, School of Physics, Universiti Sains Malaysia, 11800 USM, Penang, Malaysia; b Department of Studies in Chemistry, Mangalore University, Mangalagangotri 574 199, India; c Department of Chemistry, P. A. College of Engineering, Nadupadavu, Mangalore 574 153, India

## Abstract

In the title compound, C_31_H_20_F_4_N_4_, the pyrazole ring adopts an envelope conformation and forms a dihedral angle of 9.91 (6)° with the adjacent pyrimidine ring. The pyrimidine ring forms dihedral angles of 9.23 (6) and 2.16 (5)° with its adjacent fluoro-substituted benzene rings, whereas these angles are 88.22 (6) and 9.66 (6)° for the pyrazole ring and its adjacent benzene rings. In the crystal, mol­ecules are linked by C—H⋯F hydrogen bonds into ribbons along [01-1]. The crystal packing is further stabilized by C—H⋯π and by π–π inter­actions, with centroid–centroid distances of 3.7428 (7) and 3.7630 (6) Å.

## Related literature

For related literature, see: Calabresi *et al.* (1975 ▶); El-Hashash *et al.* (1993 ▶); Huang & Huang (2002 ▶); Marquez & Russ (2002 ▶); Townsend & Drach (2002 ▶). For related structures and background to various derivatives of 4,4′-difluoro­chalcone, see: Fun *et al.* (2010*a* ▶,*b* ▶, 2011 ▶, 2012 ▶). For the stability of the temperature controller used for data collection, see: Cosier & Glazer (1986 ▶). For ring conformations and ring puckering analysis, see: Cremer & Pople (1975 ▶). For reference bond lengths, see: Allen *et al.* (1987 ▶).
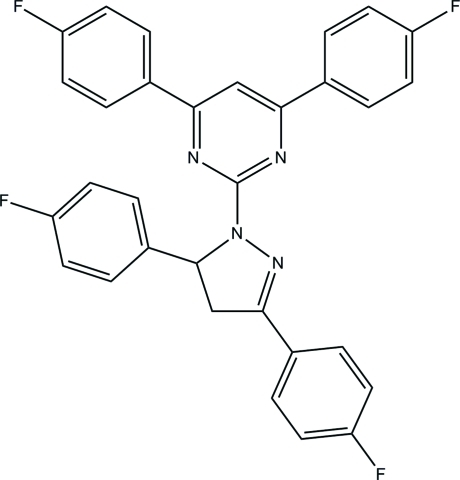


## Experimental

### 

#### Crystal data

C_31_H_20_F_4_N_4_
*M* *_r_* = 524.51
Triclinic, 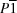

*a* = 10.1020 (1) Å
*b* = 10.1106 (1) Å
*c* = 12.3886 (1) Å
α = 104.719 (1)°
β = 98.275 (1)°
γ = 102.167 (1)°
*V* = 1169.94 (2) Å^3^
*Z* = 2
Mo *K*α radiation
μ = 0.11 mm^−1^
*T* = 100 K
0.38 × 0.26 × 0.12 mm

#### Data collection

Bruker SMART APEXII CCD area-detector diffractometer
Absorption correction: multi-scan (*SADABS*; Bruker, 2009 ▶) *T*_min_ = 0.959, *T*_max_ = 0.987
30262 measured reflections
8234 independent reflections
6443 reflections with *I* > 2σ(*I*)
*R*_int_ = 0.029

#### Refinement

*R*[*F* ^2^ > 2σ(*F* ^2^)] = 0.046
*wR*(*F* ^2^) = 0.132
*S* = 1.04
8234 reflections
352 parameters
H-atom parameters constrained
Δρ_max_ = 0.47 e Å^−3^
Δρ_min_ = −0.23 e Å^−3^

### 

Data collection: *APEX2* (Bruker, 2009 ▶); cell refinement: *SAINT* (Bruker, 2009 ▶); data reduction: *SAINT*; program(s) used to solve structure: *SHELXTL* (Sheldrick, 2008 ▶); program(s) used to refine structure: *SHELXTL*; molecular graphics: *SHELXTL*; software used to prepare material for publication: *SHELXTL* and *PLATON* (Spek, 2009 ▶).

## Supplementary Material

Crystal structure: contains datablock(s) global, I. DOI: 10.1107/S1600536812006976/fj2517sup1.cif

Structure factors: contains datablock(s) I. DOI: 10.1107/S1600536812006976/fj2517Isup2.hkl

Supplementary material file. DOI: 10.1107/S1600536812006976/fj2517Isup3.cml

Additional supplementary materials:  crystallographic information; 3D view; checkCIF report

## Figures and Tables

**Table 1:** Hydrogen-bond geometry (Å, °) *Cg*5 is the centroid of the C20–C25 benzene ring.

*D*—H⋯*A*	*D*—H	H⋯*A*	*D*⋯*A*	*D*—H⋯*A*
C2—H2*A*⋯F4^i^	0.93	2.43	3.2759 (16)	151
C31—H31*A*⋯F3^ii^	0.93	2.53	3.3208 (14)	143
C1—H1*A*⋯*Cg*5^iii^	0.93	2.98	3.7144 (14)	137
C15—H15*A*⋯*Cg*5^iv^	0.93	2.74	3.6459 (13)	166

Symmetry codes: (i) 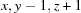; (ii) 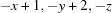; (iii) 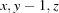; (iv) 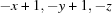.

**Fig. 1.**
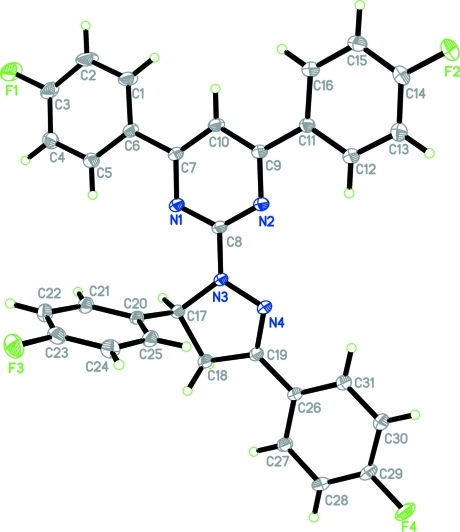
The molecular structure of the title compound with atom labels and 50% probability displacement ellipsoids.

**Fig. 2.**
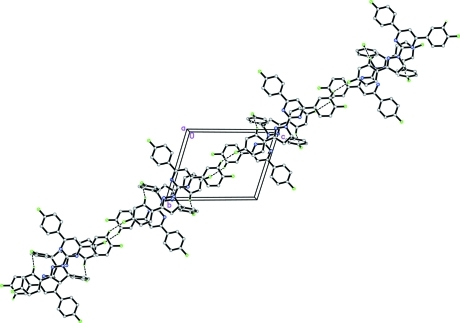
The crystal packing of the title compound. The dashed lines represent the hydrogen bonds. For clarity sake, hydrogen atoms not involved in hydrogen bonding have been omitted.

## supplementary crystallographic information

### Comment

The importance of pyrimidines and analogous compounds in pharmaceutical and biological fields is well known (Townsend & Drach, 2002). Some substituted pyrimidines and their derivatives have been reported to possess antimicrobial and antifungal activities (El-Hashash *et al.*, 1993). It has incidental antiviral activity against herpes and vaccinia infections (Calabresi *et al.*, 1975). With the development of clinically useful pyrimidine-based antitumor (Huang & Huang, 2002) and antiviral (Marquez & Russ, 2002) drugs, there has been noticeable interest in synthetic manipulations of pyrimidines. In view of the biological importance of pyrimidines and in continuation of our work on synthesis of various derivatives of 4,4'-difluoro chalcone (Fun *et al.*, 2010*a*,*b*;2011;2012), the title compound is prepared and its crystal structure is reported.

The molecular structure of the title compound is shown in Fig. 1. The pyrazole ring (N3/N4/C17–C19) adopts an envelope conformation [puckering parameters *Q* = 0.1757 (11) Å and φ = 135.8 (4)° (Cremer & Pople, 1975)] and forms a dihedral angle of 9.91 (6)° with the adjacent pyrimidine ring (N1/N2/C7–C10) (maximum deviation = 0.011 (1) Å at atom N1). The pyrimidine ring forms dihedral angles of 9.23 (6) and 2.16 (5)° with its adjacent fluoro-substituted benzene rings (C1–C6 & C11–C16, respectively), whereas for pyrazole ring these angles are 88.22 (6) and 9.66 (6)° (C20–C25 & C26–C31, respectively). In the crystal packing, the molecules are linked by intermolecular C—H···F hydrogen bonds into ribbons along [01–1]. The crystal packing is further stabilized by C–H···π interactions (Table 1), involving *Cg*5 which is the centroid of C20–C25 ring. π–π interactions are also observed with *Cg*2···*Cg*4 = 3.7428 (7) Å (symmetry code = 1-*X*,1-Y,-*Z*) and *Cg*2···*Cg*6 = 3.7630 (6) Å (symmetry code = 2-*X*,2-Y,-*Z*), where *Cg*2, *Cg*4 and *Cg*6 are the centroids of N1/N2/C7–C10, C11–C16 and C26–C31 rings, respectively.

### Experimental

A mixture of 4,4'-difluoro chalcone (2.44 g, 0.01 mol) and amino guanidine hydrochloride (0.065 g, 0.005 mol) in 25 ml e thanol was refluxed for 24 h in the presence of sodium ethoxide (2 ml). The reaction mixture was cooled to room temperature and refrigerated overnight. The solid product obtained was filtered and recrystallized from ethanol to get a yellow powder. The single crystals were grown from MDC by slow evaporation method and the yield of the compound was 49% (*m.p.*: 548 K).

### Refinement

All H atoms were positioned geometrically (C—H = 0.93, 0.97 or 0.98 Å) and refined using a riding model, with *U*_iso_(H) = 1.2*U*_eq_(C).

### Crystal data

**Table:**

C_31_H_20_F_4_N_4_	*Z* = 2
*M**_r_* = 524.51	*F*(000) = 540
Triclinic, *P*1	*D*_x_ = 1.489 Mg m^−^^3^
Hall symbol: -P 1	Mo *K*α radiation, λ = 0.71073 Å
*a* = 10.1020 (1) Å	Cell parameters from 9041 reflections
*b* = 10.1106 (1) Å	θ = 2.6–32.2°
*c* = 12.3886 (1) Å	µ = 0.11 mm^−^^1^
α = 104.719 (1)°	*T* = 100 K
β = 98.275 (1)°	Block, orange
γ = 102.167 (1)°	0.38 × 0.26 × 0.12 mm
*V* = 1169.94 (2) Å^3^	

### Data collection

**Table:**

Bruker SMART APEXII CCD area-detector diffractometer	8234 independent reflections
Radiation source: fine-focus sealed tube	6443 reflections with *I* > 2σ(*I*)
Graphite monochromator	*R*_int_ = 0.029
φ and ω scans	θ_max_ = 32.3°, θ_min_ = 2.2°
Absorption correction: multi-scan (*SADABS*; Bruker, 2009)	*h* = −15→15
*T*_min_ = 0.959, *T*_max_ = 0.987	*k* = −15→15
30262 measured reflections	*l* = −18→18

### Refinement

**Table:**

Refinement on *F*^2^	Primary atom site location: structure-invariant direct methods
Least-squares matrix: full	Secondary atom site location: difference Fourier map
*R*[*F*^2^ > 2σ(*F*^2^)] = 0.046	Hydrogen site location: inferred from neighbouring sites
*wR*(*F*^2^) = 0.132	H-atom parameters constrained
*S* = 1.04	*w* = 1/[σ^2^(*F*_o_^2^) + (0.069*P*)^2^ + 0.3191*P*] where *P* = (*F*_o_^2^ + 2*F*_c_^2^)/3
8234 reflections	(Δ/σ)_max_ < 0.001
352 parameters	Δρ_max_ = 0.47 e Å^−^^3^
0 restraints	Δρ_min_ = −0.23 e Å^−^^3^

### Special details

**Table:**

Experimental. The crystal was placed in the cold stream of an Oxford Cryosystems Cobra open-flow nitrogen cryostat (Cosier & Glazer, 1986) operating at 100.0 (1) K.
Geometry. All e.s.d.'s (except the e.s.d. in the dihedral angle between two l.s. planes) are estimated using the full covariance matrix. The cell e.s.d.'s are taken into account individually in the estimation of e.s.d.'s in distances, angles and torsion angles; correlations between e.s.d.'s in cell parameters are only used when they are defined by crystal symmetry. An approximate (isotropic) treatment of cell e.s.d.'s is used for estimating e.s.d.'s involving l.s. planes.
Refinement. Refinement of *F*^2^ against ALL reflections. The weighted *R*-factor *wR* and goodness of fit *S* are based on *F*^2^, conventional *R*-factors *R* are based on *F*, with *F* set to zero for negative *F*^2^. The threshold expression of *F*^2^ > σ(*F*^2^) is used only for calculating *R*-factors(gt) *etc*. and is not relevant to the choice of reflections for refinement. *R*-factors based on *F*^2^ are statistically about twice as large as those based on *F*, and *R*- factors based on ALL data will be even larger.

### Fractional atomic coordinates and isotropic or equivalent isotropic displacement parameters (Å^2^)

**Table:**

	*x*	*y*	*z*	*U*_iso_*/*U*_eq_	
F1	0.89250 (10)	0.66229 (9)	0.65306 (6)	0.0382 (2)	
F2	0.46323 (8)	0.14504 (8)	−0.40779 (6)	0.02907 (17)	
F3	0.41746 (8)	1.23828 (8)	0.34142 (7)	0.02823 (17)	
F4	0.85454 (8)	1.31912 (8)	−0.39439 (6)	0.02724 (17)	
N1	0.80575 (9)	0.81293 (10)	0.19025 (7)	0.01531 (16)	
N2	0.73090 (9)	0.71218 (9)	−0.01349 (8)	0.01557 (16)	
N3	0.82490 (10)	0.95255 (9)	0.07072 (7)	0.01658 (17)	
N4	0.81772 (9)	0.97309 (10)	−0.03568 (7)	0.01557 (17)	
C1	0.79155 (15)	0.54528 (13)	0.34519 (10)	0.0270 (3)	
H1A	0.7638	0.4614	0.2856	0.032*	
C2	0.82258 (16)	0.54050 (14)	0.45700 (11)	0.0333 (3)	
H2A	0.8166	0.4545	0.4728	0.040*	
C3	0.86230 (14)	0.66581 (14)	0.54355 (10)	0.0255 (2)	
C4	0.87484 (13)	0.79565 (13)	0.52391 (10)	0.0235 (2)	
H4A	0.9030	0.8790	0.5841	0.028*	
C5	0.84417 (12)	0.79855 (12)	0.41174 (10)	0.0210 (2)	
H5A	0.8523	0.8852	0.3969	0.025*	
C6	0.80148 (11)	0.67431 (11)	0.32098 (9)	0.01634 (19)	
C7	0.77219 (10)	0.68237 (11)	0.20211 (9)	0.01509 (18)	
C8	0.78434 (10)	0.81953 (11)	0.08231 (9)	0.01468 (18)	
C9	0.69487 (10)	0.58258 (11)	−0.00015 (9)	0.01464 (18)	
C10	0.71525 (11)	0.56267 (11)	0.10790 (9)	0.01625 (19)	
H10A	0.6917	0.4727	0.1169	0.019*	
C11	0.63204 (10)	0.46548 (11)	−0.10704 (9)	0.01510 (18)	
C12	0.61879 (12)	0.49503 (12)	−0.21201 (9)	0.0189 (2)	
H12A	0.6493	0.5878	−0.2137	0.023*	
C13	0.56065 (13)	0.38780 (13)	−0.31367 (10)	0.0220 (2)	
H13A	0.5510	0.4078	−0.3832	0.026*	
C14	0.51758 (12)	0.25068 (12)	−0.30883 (10)	0.0210 (2)	
C15	0.52830 (12)	0.21662 (12)	−0.20708 (10)	0.0204 (2)	
H15A	0.4980	0.1235	−0.2063	0.025*	
C16	0.58535 (11)	0.32499 (12)	−0.10650 (9)	0.0180 (2)	
H16A	0.5928	0.3041	−0.0373	0.022*	
C17	0.86637 (11)	1.08631 (11)	0.16485 (8)	0.01558 (18)	
H17A	0.9378	1.0824	0.2256	0.019*	
C18	0.92757 (11)	1.19104 (11)	0.10206 (9)	0.01707 (19)	
H18A	0.8960	1.2766	0.1209	0.020*	
H18B	1.0280	1.2159	0.1198	0.020*	
C19	0.87065 (10)	1.10600 (11)	−0.02133 (9)	0.01479 (18)	
C20	0.74267 (11)	1.12033 (11)	0.21187 (9)	0.01539 (18)	
C21	0.74268 (11)	1.14552 (12)	0.32750 (9)	0.0183 (2)	
H21A	0.8178	1.1375	0.3762	0.022*	
C22	0.63167 (12)	1.18269 (12)	0.37163 (9)	0.0207 (2)	
H22A	0.6310	1.1975	0.4487	0.025*	
C23	0.52321 (11)	1.19689 (12)	0.29813 (10)	0.0206 (2)	
C24	0.51718 (12)	1.17086 (12)	0.18210 (10)	0.0211 (2)	
H24A	0.4418	1.1796	0.1342	0.025*	
C25	0.62721 (11)	1.13136 (12)	0.13951 (9)	0.0185 (2)	
H25A	0.6247	1.1118	0.0616	0.022*	
C26	0.87168 (10)	1.16138 (11)	−0.11947 (9)	0.01481 (18)	
C27	0.92096 (11)	1.30626 (11)	−0.10361 (9)	0.01704 (19)	
H27A	0.9579	1.3672	−0.0303	0.020*	
C28	0.91544 (12)	1.36053 (12)	−0.19632 (10)	0.0193 (2)	
H28A	0.9478	1.4570	−0.1860	0.023*	
C29	0.86081 (11)	1.26735 (12)	−0.30369 (9)	0.0193 (2)	
C30	0.81118 (12)	1.12320 (12)	−0.32338 (9)	0.0197 (2)	
H30A	0.7745	1.0632	−0.3971	0.024*	
C31	0.81755 (11)	1.07049 (12)	−0.23047 (9)	0.01756 (19)	
H31A	0.7856	0.9737	−0.2419	0.021*	

### Atomic displacement parameters (Å^2^)

**Table:**

	*U*^11^	*U*^22^	*U*^33^	*U*^12^	*U*^13^	*U*^23^
F1	0.0612 (6)	0.0311 (4)	0.0157 (3)	0.0003 (4)	−0.0045 (3)	0.0118 (3)
F2	0.0366 (4)	0.0216 (4)	0.0209 (3)	0.0025 (3)	0.0030 (3)	−0.0020 (3)
F3	0.0246 (3)	0.0301 (4)	0.0304 (4)	0.0098 (3)	0.0107 (3)	0.0046 (3)
F4	0.0355 (4)	0.0302 (4)	0.0214 (3)	0.0086 (3)	0.0041 (3)	0.0180 (3)
N1	0.0177 (4)	0.0148 (4)	0.0152 (4)	0.0044 (3)	0.0035 (3)	0.0072 (3)
N2	0.0183 (4)	0.0132 (4)	0.0158 (4)	0.0036 (3)	0.0034 (3)	0.0059 (3)
N3	0.0254 (4)	0.0127 (4)	0.0124 (4)	0.0038 (3)	0.0040 (3)	0.0057 (3)
N4	0.0196 (4)	0.0151 (4)	0.0140 (4)	0.0047 (3)	0.0043 (3)	0.0071 (3)
C1	0.0418 (7)	0.0163 (5)	0.0174 (5)	−0.0014 (5)	−0.0033 (5)	0.0078 (4)
C2	0.0533 (8)	0.0191 (6)	0.0211 (5)	−0.0027 (5)	−0.0061 (5)	0.0120 (5)
C3	0.0329 (6)	0.0258 (6)	0.0155 (5)	0.0014 (5)	−0.0011 (4)	0.0105 (4)
C4	0.0335 (6)	0.0210 (5)	0.0158 (5)	0.0087 (5)	0.0019 (4)	0.0052 (4)
C5	0.0294 (5)	0.0178 (5)	0.0173 (5)	0.0081 (4)	0.0037 (4)	0.0071 (4)
C6	0.0180 (4)	0.0165 (5)	0.0152 (4)	0.0032 (4)	0.0021 (3)	0.0076 (4)
C7	0.0158 (4)	0.0154 (5)	0.0161 (4)	0.0046 (3)	0.0035 (3)	0.0078 (4)
C8	0.0164 (4)	0.0135 (4)	0.0159 (4)	0.0041 (3)	0.0038 (3)	0.0068 (4)
C9	0.0143 (4)	0.0142 (4)	0.0168 (4)	0.0042 (3)	0.0031 (3)	0.0064 (4)
C10	0.0184 (4)	0.0141 (4)	0.0170 (4)	0.0031 (4)	0.0024 (4)	0.0073 (4)
C11	0.0161 (4)	0.0138 (4)	0.0162 (4)	0.0045 (3)	0.0034 (3)	0.0053 (4)
C12	0.0237 (5)	0.0164 (5)	0.0175 (5)	0.0055 (4)	0.0041 (4)	0.0066 (4)
C13	0.0277 (5)	0.0209 (5)	0.0169 (5)	0.0066 (4)	0.0037 (4)	0.0051 (4)
C14	0.0212 (5)	0.0186 (5)	0.0196 (5)	0.0046 (4)	0.0034 (4)	0.0004 (4)
C15	0.0209 (5)	0.0153 (5)	0.0248 (5)	0.0035 (4)	0.0055 (4)	0.0060 (4)
C16	0.0188 (4)	0.0159 (5)	0.0205 (5)	0.0045 (4)	0.0043 (4)	0.0070 (4)
C17	0.0194 (4)	0.0132 (4)	0.0129 (4)	0.0024 (4)	0.0016 (3)	0.0042 (3)
C18	0.0210 (5)	0.0141 (4)	0.0149 (4)	0.0014 (4)	0.0030 (4)	0.0052 (4)
C19	0.0169 (4)	0.0140 (4)	0.0142 (4)	0.0033 (3)	0.0033 (3)	0.0060 (4)
C20	0.0187 (4)	0.0121 (4)	0.0142 (4)	0.0017 (3)	0.0020 (3)	0.0046 (3)
C21	0.0213 (5)	0.0188 (5)	0.0143 (4)	0.0041 (4)	0.0025 (4)	0.0052 (4)
C22	0.0243 (5)	0.0201 (5)	0.0165 (5)	0.0044 (4)	0.0054 (4)	0.0040 (4)
C23	0.0197 (5)	0.0167 (5)	0.0245 (5)	0.0040 (4)	0.0065 (4)	0.0042 (4)
C24	0.0198 (5)	0.0212 (5)	0.0212 (5)	0.0050 (4)	0.0012 (4)	0.0064 (4)
C25	0.0220 (5)	0.0181 (5)	0.0145 (4)	0.0037 (4)	0.0020 (4)	0.0056 (4)
C26	0.0161 (4)	0.0142 (4)	0.0156 (4)	0.0038 (3)	0.0038 (3)	0.0069 (4)
C27	0.0193 (4)	0.0156 (5)	0.0172 (4)	0.0035 (4)	0.0037 (4)	0.0073 (4)
C28	0.0220 (5)	0.0169 (5)	0.0215 (5)	0.0042 (4)	0.0053 (4)	0.0104 (4)
C29	0.0208 (5)	0.0235 (5)	0.0188 (5)	0.0076 (4)	0.0046 (4)	0.0138 (4)
C30	0.0238 (5)	0.0208 (5)	0.0152 (4)	0.0057 (4)	0.0030 (4)	0.0070 (4)
C31	0.0209 (5)	0.0158 (5)	0.0164 (4)	0.0040 (4)	0.0038 (4)	0.0061 (4)

### Geometric parameters (Å, º)

**Table:**

F1—C3	1.3582 (12)	C13—H13A	0.9300
F2—C14	1.3561 (13)	C14—C15	1.3844 (16)
F3—C23	1.3577 (13)	C15—C16	1.3852 (16)
F4—C29	1.3538 (11)	C15—H15A	0.9300
N1—C7	1.3429 (13)	C16—H16A	0.9300
N1—C8	1.3445 (12)	C17—C20	1.5193 (15)
N2—C8	1.3386 (14)	C17—C18	1.5458 (14)
N2—C9	1.3428 (13)	C17—H17A	0.9800
N3—C8	1.3712 (13)	C18—C19	1.5090 (14)
N3—N4	1.3798 (11)	C18—H18A	0.9700
N3—C17	1.4777 (14)	C18—H18B	0.9700
N4—C19	1.2929 (13)	C19—C26	1.4625 (13)
C1—C2	1.3909 (16)	C20—C21	1.3897 (14)
C1—C6	1.3980 (15)	C20—C25	1.4076 (14)
C1—H1A	0.9300	C21—C22	1.3955 (16)
C2—C3	1.3738 (19)	C21—H21A	0.9300
C2—H2A	0.9300	C22—C23	1.3769 (16)
C3—C4	1.3784 (17)	C22—H22A	0.9300
C4—C5	1.3886 (15)	C23—C24	1.3839 (16)
C4—H4A	0.9300	C24—C25	1.3864 (16)
C5—C6	1.3938 (16)	C24—H24A	0.9300
C5—H5A	0.9300	C25—H25A	0.9300
C6—C7	1.4861 (14)	C26—C27	1.3966 (14)
C7—C10	1.3975 (15)	C26—C31	1.4015 (15)
C9—C10	1.3964 (13)	C27—C28	1.3921 (14)
C9—C11	1.4842 (15)	C27—H27A	0.9300
C10—H10A	0.9300	C28—C29	1.3763 (16)
C11—C12	1.4008 (14)	C28—H28A	0.9300
C11—C16	1.4013 (14)	C29—C30	1.3824 (16)
C12—C13	1.3898 (16)	C30—C31	1.3848 (14)
C12—H12A	0.9300	C30—H30A	0.9300
C13—C14	1.3822 (16)	C31—H31A	0.9300
			
C7—N1—C8	115.54 (9)	C11—C16—H16A	119.4
C8—N2—C9	116.12 (9)	N3—C17—C20	111.40 (8)
C8—N3—N4	121.04 (9)	N3—C17—C18	100.36 (8)
C8—N3—C17	125.56 (8)	C20—C17—C18	112.75 (8)
N4—N3—C17	113.14 (8)	N3—C17—H17A	110.6
C19—N4—N3	107.85 (8)	C20—C17—H17A	110.6
C2—C1—C6	121.00 (11)	C18—C17—H17A	110.6
C2—C1—H1A	119.5	C19—C18—C17	101.79 (8)
C6—C1—H1A	119.5	C19—C18—H18A	111.4
C3—C2—C1	118.41 (11)	C17—C18—H18A	111.4
C3—C2—H2A	120.8	C19—C18—H18B	111.4
C1—C2—H2A	120.8	C17—C18—H18B	111.4
F1—C3—C2	118.88 (10)	H18A—C18—H18B	109.3
F1—C3—C4	118.40 (11)	N4—C19—C26	120.47 (9)
C2—C3—C4	122.71 (10)	N4—C19—C18	113.62 (8)
C3—C4—C5	118.12 (11)	C26—C19—C18	125.90 (9)
C3—C4—H4A	120.9	C21—C20—C25	118.57 (10)
C5—C4—H4A	120.9	C21—C20—C17	120.88 (9)
C4—C5—C6	121.40 (10)	C25—C20—C17	120.52 (9)
C4—C5—H5A	119.3	C20—C21—C22	120.97 (10)
C6—C5—H5A	119.3	C20—C21—H21A	119.5
C5—C6—C1	118.34 (10)	C22—C21—H21A	119.5
C5—C6—C7	119.64 (9)	C23—C22—C21	118.33 (10)
C1—C6—C7	121.99 (10)	C23—C22—H22A	120.8
N1—C7—C10	121.61 (9)	C21—C22—H22A	120.8
N1—C7—C6	115.61 (9)	F3—C23—C22	118.54 (10)
C10—C7—C6	122.78 (9)	F3—C23—C24	118.54 (10)
N2—C8—N1	127.67 (9)	C22—C23—C24	122.93 (11)
N2—C8—N3	117.20 (9)	C23—C24—C25	117.86 (10)
N1—C8—N3	115.12 (9)	C23—C24—H24A	121.1
N2—C9—C10	121.18 (10)	C25—C24—H24A	121.1
N2—C9—C11	115.31 (9)	C24—C25—C20	121.28 (10)
C10—C9—C11	123.50 (9)	C24—C25—H25A	119.4
C9—C10—C7	117.85 (9)	C20—C25—H25A	119.4
C9—C10—H10A	121.1	C27—C26—C31	119.09 (9)
C7—C10—H10A	121.1	C27—C26—C19	120.48 (9)
C12—C11—C16	118.56 (10)	C31—C26—C19	120.37 (9)
C12—C11—C9	119.53 (9)	C28—C27—C26	120.75 (10)
C16—C11—C9	121.91 (9)	C28—C27—H27A	119.6
C13—C12—C11	120.98 (10)	C26—C27—H27A	119.6
C13—C12—H12A	119.5	C29—C28—C27	118.12 (10)
C11—C12—H12A	119.5	C29—C28—H28A	120.9
C14—C13—C12	118.31 (10)	C27—C28—H28A	120.9
C14—C13—H13A	120.8	F4—C29—C28	118.58 (10)
C12—C13—H13A	120.8	F4—C29—C30	118.33 (10)
F2—C14—C13	118.70 (10)	C28—C29—C30	123.09 (10)
F2—C14—C15	118.61 (10)	C29—C30—C31	118.24 (10)
C13—C14—C15	122.68 (11)	C29—C30—H30A	120.9
C14—C15—C16	118.22 (10)	C31—C30—H30A	120.9
C14—C15—H15A	120.9	C30—C31—C26	120.72 (10)
C16—C15—H15A	120.9	C30—C31—H31A	119.6
C15—C16—C11	121.23 (10)	C26—C31—H31A	119.6
C15—C16—H16A	119.4		
			
C8—N3—N4—C19	−175.75 (9)	C13—C14—C15—C16	−0.36 (17)
C17—N3—N4—C19	9.72 (12)	C14—C15—C16—C11	−0.41 (16)
C6—C1—C2—C3	0.5 (2)	C12—C11—C16—C15	0.57 (16)
C1—C2—C3—F1	179.84 (13)	C9—C11—C16—C15	−179.29 (10)
C1—C2—C3—C4	−1.1 (2)	C8—N3—C17—C20	−71.63 (12)
F1—C3—C4—C5	179.83 (11)	N4—N3—C17—C20	102.61 (9)
C2—C3—C4—C5	0.8 (2)	C8—N3—C17—C18	168.78 (10)
C3—C4—C5—C6	0.17 (19)	N4—N3—C17—C18	−16.98 (11)
C4—C5—C6—C1	−0.74 (18)	N3—C17—C18—C19	16.42 (10)
C4—C5—C6—C7	−178.88 (10)	C20—C17—C18—C19	−102.19 (10)
C2—C1—C6—C5	0.4 (2)	N3—N4—C19—C26	−177.14 (9)
C2—C1—C6—C7	178.49 (12)	N3—N4—C19—C18	2.85 (12)
C8—N1—C7—C10	−1.71 (14)	C17—C18—C19—N4	−13.07 (12)
C8—N1—C7—C6	177.45 (9)	C17—C18—C19—C26	166.92 (10)
C5—C6—C7—N1	7.77 (14)	N3—C17—C20—C21	125.54 (10)
C1—C6—C7—N1	−170.30 (11)	C18—C17—C20—C21	−122.51 (10)
C5—C6—C7—C10	−173.07 (10)	N3—C17—C20—C25	−56.48 (12)
C1—C6—C7—C10	8.86 (17)	C18—C17—C20—C25	55.47 (13)
C9—N2—C8—N1	−0.24 (16)	C25—C20—C21—C22	−0.77 (16)
C9—N2—C8—N3	178.53 (9)	C17—C20—C21—C22	177.25 (10)
C7—N1—C8—N2	1.70 (15)	C20—C21—C22—C23	−1.41 (17)
C7—N1—C8—N3	−177.10 (9)	C21—C22—C23—F3	−177.53 (10)
N4—N3—C8—N2	−3.49 (14)	C21—C22—C23—C24	2.47 (18)
C17—N3—C8—N2	170.33 (9)	F3—C23—C24—C25	178.77 (10)
N4—N3—C8—N1	175.44 (9)	C22—C23—C24—C25	−1.23 (18)
C17—N3—C8—N1	−10.74 (15)	C23—C24—C25—C20	−1.09 (17)
C8—N2—C9—C10	−1.20 (14)	C21—C20—C25—C24	2.06 (16)
C8—N2—C9—C11	178.28 (9)	C17—C20—C25—C24	−175.97 (10)
N2—C9—C10—C7	1.11 (15)	N4—C19—C26—C27	175.99 (10)
C11—C9—C10—C7	−178.34 (9)	C18—C19—C26—C27	−4.00 (16)
N1—C7—C10—C9	0.45 (15)	N4—C19—C26—C31	−1.19 (15)
C6—C7—C10—C9	−178.66 (9)	C18—C19—C26—C31	178.82 (10)
N2—C9—C11—C12	2.11 (14)	C31—C26—C27—C28	0.63 (16)
C10—C9—C11—C12	−178.42 (10)	C19—C26—C27—C28	−176.58 (10)
N2—C9—C11—C16	−178.03 (9)	C26—C27—C28—C29	−0.32 (16)
C10—C9—C11—C16	1.44 (16)	C27—C28—C29—F4	179.78 (10)
C16—C11—C12—C13	0.02 (16)	C27—C28—C29—C30	0.15 (17)
C9—C11—C12—C13	179.89 (10)	F4—C29—C30—C31	−179.93 (10)
C11—C12—C13—C14	−0.74 (17)	C28—C29—C30—C31	−0.31 (17)
C12—C13—C14—F2	−178.73 (10)	C29—C30—C31—C26	0.62 (16)
C12—C13—C14—C15	0.93 (18)	C27—C26—C31—C30	−0.79 (16)
F2—C14—C15—C16	179.29 (10)	C19—C26—C31—C30	176.43 (10)

### Hydrogen-bond geometry (Å, º)

Cg5 is the centroid of the C20–C25 benzene ring.

**Table:**

*D*—H···*A*	*D*—H	H···*A*	*D*···*A*	*D*—H···*A*
C2—H2*A*···F4^i^	0.93	2.43	3.2759 (16)	151
C31—H31*A*···F3^ii^	0.93	2.53	3.3208 (14)	143
C1—H1*A*···*Cg*5^iii^	0.93	2.98	3.7144 (14)	137
C15—H15*A*···*Cg*5^iv^	0.93	2.74	3.6459 (13)	166

Symmetry codes: (i) *x*, *y*−1, *z*+1; (ii) −*x*+1, −*y*+2, −*z*; (iii) *x*, *y*−1, *z*; (iv) −*x*+1, −*y*+1, −*z*.
